# Using a Genetically Encoded Sensor to Identify Inhibitors of *Toxoplasma gondii* Ca^2+^ Signaling*[Fn FN2]

**DOI:** 10.1074/jbc.M115.703546

**Published:** 2016-03-01

**Authors:** Saima M. Sidik, Miryam A. Hortua Triana, Aditya S. Paul, Majida El Bakkouri, Caroline G. Hackett, Fanny Tran, Nicholas J. Westwood, Raymond Hui, William J. Zuercher, Manoj T. Duraisingh, Silvia N. J. Moreno, Sebastian Lourido

**Affiliations:** From the ‡ Whitehead Institute for Biomedical Research, Cambridge, Massachusetts 02142,; the § Center for Tropical and Emerging Global Diseases, Department of Cellular Biology, University of Georgia, Athens, Georgia 30602,; the ¶ Department of Immunology and Infectious Diseases, Harvard T. H. Chan School of Public Health, Boston, Massachusetts 02115,; the ‖ Structural Genomics Consortium, University of Toronto, Toronto, Ontario M5G 1L7, Canada,; the ** School of Chemistry and Biomedical Sciences Research Complex, University of St. Andrews and EaStCHEM, North Haugh, St. Andrews, Fife KY16 9ST, Scotland, United Kingdom, and; the ‡‡ Division of Chemical Biology and Medicinal Chemistry, UNC Eshelman School of Pharmacy, University of North Carolina, Chapel Hill, North Carolina 27599

**Keywords:** calcium, calcium intracellular release, drug screening, parasitology, protein kinase G (PKG), signal transduction

## Abstract

The life cycles of apicomplexan parasites progress in accordance with fluxes in cytosolic Ca^2+^. Such fluxes are necessary for events like motility and egress from host cells. We used genetically encoded Ca^2+^ indicators (GCaMPs) to develop a cell-based phenotypic screen for compounds that modulate Ca^2+^ signaling in the model apicomplexan *Toxoplasma gondii*. In doing so, we took advantage of the phosphodiesterase inhibitor zaprinast, which we show acts in part through cGMP-dependent protein kinase (protein kinase G; PKG) to raise levels of cytosolic Ca^2+^. We define the pool of Ca^2+^ regulated by PKG to be a neutral store distinct from the endoplasmic reticulum. Screening a library of 823 ATP mimetics, we identify both inhibitors and enhancers of Ca^2+^ signaling. Two such compounds constitute novel PKG inhibitors and prevent zaprinast from increasing cytosolic Ca^2+^. The enhancers identified are capable of releasing intracellular Ca^2+^ stores independently of zaprinast or PKG. One of these enhancers blocks parasite egress and invasion and shows strong antiparasitic activity against *T. gondii*. The same compound inhibits invasion of the most lethal malaria parasite, *Plasmodium falciparum*. Inhibition of Ca^2+^-related phenotypes in these two apicomplexan parasites suggests that depletion of intracellular Ca^2+^ stores by the enhancer may be an effective antiparasitic strategy. These results establish a powerful new strategy for identifying compounds that modulate the essential parasite signaling pathways regulated by Ca^2+^, underscoring the importance of these pathways and the therapeutic potential of their inhibition.

## Introduction

Apicomplexan parasites, such as *Toxoplasma gondii* and *Plasmodium* spp., the causative agents of toxoplasmosis and malaria, require changes in cytosolic Ca^2+^ concentrations to egress from host cells and move within the infected organism (1–6). These pathways therefore hold tremendous therapeutic potential, not only due to their importance in parasite biology but because of their divergence from similar pathways in host cells. Compounds targeting Ca^2+^ signaling in parasites have been shown to be effective antiparasitics (discussed below). However, few of the molecules involved in regulating Ca^2+^ homeostasis and signaling have been identified in parasites, and their interplay is only evident in live cells. This has created a need for new methods to study Ca^2+^ signaling pathways in apicomplexan parasites, with the hope of defining the essential components and identifying novel inhibitors.

The mechanisms for Ca^2+^ entry into the cytoplasm and the physiologically relevant sources of Ca^2+^ remain poorly defined in apicomplexan parasites. Ca^2+^ can be mobilized from the parasite's intracellular stores, or it can be drawn from the environment. Current evidence points toward intracellular stores being sufficient for parasites to move between cells (6–8), although virulence of *T. gondii* is enhanced by extracellular Ca^2+^ (9). The best studied of these intracellular stores is the endoplasmic reticulum (ER).^2^ This organelle is a highly networked, dynamic structure (10) that has been shown to constitute multiple spatially independent Ca^2+^ stores in some cell types (11). Such compartmentalization has also been hypothesized to occur in *T. gondii* (4). Mammalian cells store Ca^2+^ in endosomes, lysosomes (12), and the Golgi (13), in addition to the ER (14). Some alveolates, like *Paramecium*, additionally contain a network of alveolar sacs that sequester Ca^2+^ in an ATP-dependent manner, with physiologically relevant affinities (15, 16). Whether the inner membrane complex, an apicomplexan structure homologous to alveolar sacs, also stores Ca^2+^ remains to be determined. Like many other eukaryotes, apicomplexans also possess acidic vacuoles known as acidocalcisomes that contain Ca^2+^ in complex with pyrophosphate and polyphosphates. This Ca^2+^ can be released pharmacologically, but the function of acidocalcisomes remains unclear in apicomplexans (17). A final acidic Ca^2+^ store described in *T. gondii* is the plantlike vacuole or vacuolar compartment. The plantlike vacuole is an acidic organelle that releases Ca^2+^ upon treatment with l-phenylalanine-naphthylamide (GPN), which in other systems causes ion leakage from lysosomal compartments (18). Although implicated in ionic homeostasis, these phenotypes have not been linked to the plantlike vacuole's function as a Ca^2+^ store (18). Due to the lack of characterized regulatable Ca^2+^ channels, it remains an open question which of these Ca^2+^ sources are involved in parasite motility and invasion.

Recent evidence suggests that PKG may play a role in regulating parasite Ca^2+^. In *Plasmodium berghei*, PKG has been proposed to influence Ca^2+^ homeostasis, thereby regulating egress from host cells. Activation of PKG leads to change in the levels of the lipid precursors of inositol 1,4,5-triphosphate (IP_3_). This has been hypothesized to increase IP_3_, which causes release of Ca^2+^ from the ER through binding to the IP_3_ receptor, although such a channel remains to be identified in apicomplexan parasites (19). PKG is also known to regulate egress in *T. gondii*, although its effect on parasite Ca^2+^ has not been determined (20). In both parasites, PKG can be pharmacologically activated using the mammalian phosphodiesterase inhibitor zaprinast (20, 21). In *Plasmodium*, zaprinast treatment leads to an increase in cyclic GMP levels that presumably activates PKG (22). Further characterization of zaprinast's mechanism of action could therefore shed light on Ca^2+^ signaling.

Targeting the downstream effectors of Ca^2+^ signaling has been shown to hold therapeutic value against *T. gondii*. Compounds targeting Ca^2+^-dependent protein kinase 1, a regulator of egress and invasion, reduce proliferation in cell culture and cyst burden in the brains of *T. gondii*-infected mice (23, 24). Drugs targeting Ca^2+^-related processes are appealing not only because of this historical success but also because many proteins involved in parasite Ca^2+^ signaling are sufficiently divergent from their mammalian counterparts to enable the design of drugs with minimal off-target effects. For example, protein kinase G (PKG), which regulates egress, invasion, and motility in *T. gondii* and *Plasmodium* spp. (19, 20, 25, 26), is sufficiently different from mammalian PKG to be selectively inhibited (27). Similarly, the Ca^2+^-dependent protein kinases lack homologues in mammalian cells (28), making them attractive drug targets.

In this study, we use both chemical and genetic Ca^2+^ indicators to define the regulatory circuits that mediate Ca^2+^ release in *T. gondii* and identify small molecules that modulate this process. We determine the effect of *T. gondii* PKG on cytosolic Ca^2+^ following treatment with zaprinast and characterize the source of the Ca^2+^ released in this process as a neutral store distinct from the ER. Using genetically encoded Ca^2+^ indicators recently established in *T. gondii* (29), we develop a cell-based phenotypic screen that allows us to monitor Ca^2+^ signaling in live cells without the technical challenges of conventional chemical Ca^2+^ indicators. Using this platform, we have been able to identify, in an unbiased manner, compounds that interfere with Ca^2+^ signaling. In contrast to enzyme-based assays, this system enables us to probe a broader swath of parasite biology. Recent analysis indicates that such phenotypic screens are more likely to lead to clinically approved drugs than the far more prevalent molecular target-based approaches (30). Our screen identified two novel PKG inhibitors that abrogate the effect of zaprinast, as well as two compounds that increase cytosolic Ca^2+^ through an independent pathway. From the latter, one compound blocks invasion of both *T. gondii* and *Plasmodium falciparum*. Our results demonstrate the potential of this strategy to explore parasite Ca^2+^ signaling and identify new compounds with antiparasitic potential against multiple apicomplexan parasites.

## Experimental Procedures

### 

#### 

##### Strain Construction and Maintenance

Recombinant human *T. gondii* strain RH parasites were maintained in human foreskin fibroblasts (HFFs) grown in DMEM supplemented with 10% heat-inactivated fetal bovine serum (FBS) and 10 μg/ml gentamicin. PKG-T and PKG-M alleles were constructed as described previously (31). GCaMP5 was amplified from pCMV-GCaMP5G (32) with primers containing NsiI and PacI restriction sites (forward primer, 5′-gcg atg cat cct ttt tcg aca aaa tgg gtt ctc atc atc atc atc atc; reverse primer, 5′-gcg tta att aat cac ttc gct gtc atc att tg) and cloned directionally, replacing the *CAT* gene in *pSAG1/2-CAT* (33) to generate *pSAG1-GCaMP5*. Recombinant human parasites were co-transfected with *pSAG1-GCaMP5* and *pSAG1/2-CAT* and selected with chloramphenicol (40 μm), and clones were isolated by limiting dilution. The GCaMP6f strains was similarly derived, as described previously (29). Both GCaMP strains were maintained under selection to prevent loss of the transgene. The GFP-expressing strain was kindly provided by Jeroen P. J. Saeij (34).

##### Store Activation and Cpd1 Inhibition Experiments with GCaMP6f

GCaMP6f-expressing *T. gondii* were suspended at 2 × 10^7^ parasites/ml in basal Ca^2+^ buffer (140 mm NaCl, 10 mm potassium gluconate, 2.7 mm MgSO_4_, 2 mm glucose, 250 μm EGTA, 85 μm CaCl_2_, 10 mm HEPES, pH 7.3) or extracellular Ca^2+^ buffer (140 mm NaCl, 10 mm potassium gluconate, 2.7 mm MgSO_4_, 2 mm glucose, 1 mm CaCl_2_, 10 mm HEPES, pH 7.3), supplemented with 1% FBS when noted. For the Cpd1 inhibition experiments, parasites were suspended in Ringer's solution (115 mm NaCl, 3 mm KCl, 2 mm CaCl_2_, 1 mm MgCl_2_, 3 mm NaH_2_PO_4_, 10 mm HEPES, 10 mm glucose, 1% FBS). In all cases, 100 μl of suspended parasites were applied to each well of a Cell Carrier 96-well plate (PerkinElmer Life Sciences). For store activation experiments, parasites were incubated on ice for 5 min before the addition of Enh1 or Enh2 (10 μm final concentration), zaprinast (100 μm final), or DMSO (0.3% final) suspended in the same buffers as the parasites to which they were added. To examine the effect of Cpd1 on zaprinast or Enh1 treatment of GCaMP6-expressing parasites, fluorescence was recorded for 100 s before adding Cpd1 to a concentration of 1.2 μm. Fluorescence was recorded for an additional 300 s before treatment with 10 μm Enh1 or 100 μm zaprinast. Parasites were incubated on ice for 5 min before the addition of zaprinast to slow down the response and facilitate observation of peak fluorescence. Fluorescence was read with an excitation wavelength of 485 nm and an emission wavelength of 528 nm every 10 s in a BioTek Cytation 3. The assay plate was shaken for 1 s before each read.

##### Compound Screen

GCaMP5-expressing parasites were suspended in Ringer's solution at 4 × 10^7^ parasites/ml, and 50 μl of parasites were applied to each well of a 96-well plate (Costar, catalog no. 3631). Parasites were treated with compounds from the GSK libraries PKIS and PKIS2 at 13.3 μm or 1.33% DMSO as a vehicle control and then incubated at 37 °C with 5% CO_2_ for 10 min. Parasites were then treated with 100 μm zaprinast or 0.1% DMSO as a vehicle control and incubated at 37 °C with 5% CO_2_ for an additional 4 min before measuring fluorescence with excitation and emission wavelengths of 485 and 525 nm, respectively, on a SpectraMax M3 (Molecular Devices). Basal fluorescence, measured from untreated parasites, was subtracted from all values, and results were expressed as -fold change from parasites treated only with zaprinast. *Z*′ factors were determined from (i) parasites treated with zaprinast *versus* untreated parasites and (ii) parasites treated with a final concentration of 1 μm Cpd2 or a vehicle control followed by zaprinast, using the formula, *Z*′ = 1 − 3(σ_p_ + σ_n_)/(|μ_p_ − μ_n_|), where σ_p_ and σ_n_ indicate S.D. values of positive and negative controls, and μ_p_ and μ_n_ indicate the means of positive and negative controls, respectively.

##### In Vitro PKG Assays

Recombinant *T. gondii* PKG was expressed using a baculovirus system. Synthetic DNA coding for the protein was amplified and subcloned into the pFBOH-MHL vector, which confers an N-terminal His_6_ tag with a tobacco etch virus cleavage site. The resulting plasmid was transformed into DH10Bac^TM^ *Escherichia coli* competent cells to produce recombinant viral DNA. P3 viral stocks were used to infect Sf9 insect cells grown in HyQ SFX insect serum-free medium (Thermo Fisher Scientific). The culture was incubated at 27 °C and shaken at 100 rpm. After 60–72 h, the cells were harvested. The His-tagged *T. gondii* PKG samples were purified by affinity chromatography and size exclusion chromatography using an ÄKTAxpress system equipped with a Superdex^TM^ 200 10/300 column (GE Healthcare, Mississauga, Canada). *In vitro* kinase assays were performed using a PKG assay kit (CycLex) as per the manufacturer's instructions. Inhibitors were tested against 6.76 nm recombinant PKG, which was active within the linear range of the assay.

##### Structural Analysis

Structures of various protein kinases in complex with pyrazolopyridazine (PP) and oxindole derivatives were obtained from the RCSB Protein Data Bank (35) and aligned on the basis of their kinase domains. For the PP analysis, the CDK2 structures 3EID and 3EJ1 (36) and the p38 MAPK structure 3GCP (37) were aligned to the ERK2 structure 1WZY (38). For the oxindole analysis, the PDK1 structures 2PE0 and 2PE2 (39) and the Alk5 structure 2X7O were aligned to the Nek2 structure 2JAV (40).

##### Lactate Dehydrogenase Release Egress Assays

Confluent HFF monolayers in 96-well plates were infected with 5 × 10^4^ parasites/well. The HFF monolayer was washed once with Ringer's solution ∼18 h later, and 50 μl of Ringer's solution was applied to each well. Drugs suspended in Ringer's solution were applied at 1.33 times the indicated concentrations. 1.33% DMSO in Ringer's solution was used as a vehicle control. Cells were incubated at 37 °C with 5% CO_2_ for 20 min, stimulated with 500 μm zaprinast or 0.5% DMSO (vehicle), and incubated again for an additional 5 min. The cells were centrifuged at 400 × *g* for 5 min, before collecting 50 μl of each supernatant. The lactate dehydrogenase levels in the supernatant samples were quantified using the CytoTox 96 cytotoxicity assay (Promega) as per the manufacturer's instructions.

##### Plaque Assays

*T. gondii* parasites were suspended in growth medium supplemented with Enh1, zaprinast, or DMSO alone at the indicated concentrations. The amount of DMSO in all treatment groups was normalized to 0.1%. Parasites were incubated at 37 °C with 5% CO_2_ for 20 min before infecting HFF monolayers in 6-well plates using 3 ml carrying 100 parasites/well. Medium containing the highest concentration of each drug was also applied to HFF cells in the absence of parasites to assess the effects of the drugs on host cell viability. The parasites were allowed to plaque for 8 days before fixing with 70% ethanol and staining with 0.1% crystal violet.

##### Lytic Assays

Drugs were suspended at twice the indicated concentrations in growth medium and mixed with an equal volume of parasites at an initial concentration of 10^6^ parasites/ml. The maximum concentration of DMSO was added as a vehicle control: 1% DMSO in comparisons with zaprinast and 0.013% in comparisons with Enh1. Parasites were preincubated with the compounds for 20 min at 37 °C with 5% CO_2_, and then 200 μl/well (10^5^ parasites) was added to host cell monolayers in 96-well plates. Assay plates were incubated at 37 °C with 5% CO_2_ for 3 days and then fixed in 70% ethanol and stained with 0.1% crystal violet. Absorbance at 590 nm was read as a measure of host cell lysis.

##### Cell-wounding Assays

Parasites suspended in Ringer's solution at 5 × 10^5^ parasites/ml were pretreated with varying concentrations of zaprinast (as indicated) for 20 min at 37 °C with 5% CO_2_. 10^5^ parasites/well were then applied to confluent host cell monolayers, and plates were centrifuged at 290 × *g* for 5 min. Following 1 h at 37 °C with 5% CO_2_, plates were centrifuged again at 500 × *g* for 5 min. 50 μl of supernatant were collected from each well, and lactate dehydrogenase was quantified using the CytoTox 96 cytotoxicity assay (Promega).

##### Video Microscopy Egress Assays

Host cell monolayers in Cell Carrier 96-well plates (PerkinElmer Life Sciences) were infected with 5 × 10^4^ GFP-expressing parasites/well. 18 h postinfection, the medium was exchanged for Ringer's solution, and the intracellular parasites were treated with 10 μm Enh1, 500 μm zaprinast, or 0.5% DMSO. Images were acquired every 10 s for 30 min. To measure the effect of Enh1 on zaprinast and A23187-induced egress, the procedure was repeated, adding either 12.5 μm Enh1 or 0.13% DMSO and imaging for 10 min, before stimulation with 500 μm zaprinast or 1 μm A23187 (Calbiochem) and imaging for an additional 10 min. In all cases, images were acquired with a ×4 objective on a Cytation3 reader (BioTek) using excitation and emission wavelengths of 485 and 528 nm, respectively. Intact vacuoles were defined as objects of at least 78 μm^2^ with a circularity of at least 0.5, as determined in Fiji after default thresholding (41).

##### Video Microscopy

Parasites expressing GCaMP5 were transfected with *p30-DsRed* and used to infect host cell monolayers in 3-cm glass bottom dishes (MatTek). Approximately 20 h after infection, medium was exchanged for Ringer's solution, and parasites were imaged on a Nikon Eclipse Ti epifluorescence microscope, equipped with an enclosure heated to 37 °C. Images were acquired every 5 s for 10 min following the addition of zaprinast to a final concentration of 100 μm. To quantify changes in fluorescence, videos were analyzed in Fiji to measure the average fluorescence intensity in specific circular regions of interest 10 μm in diameter. To examine the effects of zaprinast and Enh1 on mammalian cells, 6 × 10^3^ HeLa cells were seeded in each well of a Cell Carrier 96-well plate. Approximately 24 h later, HeLa cells were transfected with 100 ng of the *pCMV-R-GECO* (42) using Fugene (Promega) as per the manufacturer's instructions. Cells were washed in Ringer's solution and then treated with 10 μm Enh1, 500 μm zaprinast, or 2 μm A23187. Images were acquired every 12 s for 30 min on a Cytation3 reader (BioTek) using an excitation wavelength of 531 and an emission wavelength of 593. Higher resolution videos were similarly acquired from HeLa cells seeded in 3-cm glass bottom dishes (MatTek) and transfected with 200 ng of *pCMV-R-GECO* per dish. Cells were treated with either 500 μm zaprinast or 10 μm Enh1 in Ringer's solution and imaged every 250 ms using a Nikon Eclipse Ti microscope. Videos were analyzed in Fiji using default thresholding for the red channel to determine the mean gray value in each slice for individual cells. Kymographs were constructed from videos of intracellular GCaMP6f-expressing parasites, following the same treatment as for the video microscopy egress assays, except half as many parasites were used to infect the monolayers, and a ×20 objective was used to acquire the images. Regions of interest were defined by outlining parasites in Fiji and measuring the mean fluorescence of each region over time.

##### Fura-2 Recordings

Tachyzoite loading with Fura-2/AM was done as described previously (17). Briefly, freshly lysed parasites were washed twice with buffer A (116 mm NaCl, 5.4 mm KCl, 0.8 mm MgSO_4_, 5.5 mm d-glucose, and 50 mm HEPES, pH 7.4) and resuspended to a final density of 1 × l0^9^ parasites/ml in loading buffer (buffer A plus 1.5% sucrose and 5 μm Fura-2/AM). The suspension was incubated for 26 min at 26 °C with mild agitation. Subsequently, the parasites were washed twice with buffer A to remove extracellular dye, resuspended to a final density of 1 × 10^9^ parasites/ml in buffer A, and kept in ice. Parasites are viable for a few hours under these conditions. For fluorescence measurements, 2 × 10^7^ parasites/ml were placed in a cuvette with 2.5 ml of Ringer's solution. Fluorescence measurements were done in a thermostatically controlled Hitachi F-7000 spectrofluorometer using the Fura-2 conditions for excitation (340 and 380 nm) and emission (510 nm). The Fura-2 fluorescence response to Ca^2+^ was calibrated from the ratio of 340/380-nm fluorescence values after subtraction of the background fluorescence of the cells at 340 and 380 nm, as described previously (43). The Ca^2+^ release rate is the change in Ca^2+^ concentration during the initial 20 s after the addition of compound.

##### Chemical Susceptibility of P. falciparum Egress and Erythrocyte Invasion

Blood stage *P. falciparum* parasites of strain 3D7 were obtained from the Walter and Eliza Hall Institute (Melbourne, Australia) and cultured as described previously (44). Parasites were maintained in O^+^ human erythrocytes (Research Blood Components, Boston, MA), at 2% hematocrit, in RPMI 1640 supplemented with HEPES (25 mm), hypoxanthine (50 mg/liter), sodium bicarbonate (2.42 mm), and Albumax (4.31 mg/ml). Cultures were incubated at 37 °C in microaerophilic atmospheric conditions (1% O_2_, 5% CO_2_, 94% N_2_) within modular incubator chambers.

To test the specific effects of Enh1 on egress of parasites from schizonts as well as erythrocyte invasion by liberated merozoites, mature schizont stage parasites were purified from 4 ml of blood stage culture (10–20% hematocrit) by centrifugation (930 × *g*, 15 min, low acceleration and deceleration) on 4 ml of a 60% Percoll cushion (45). Schizonts were retrieved from the medium supernatant-Percoll interface. After at least three washes in excess volumes of RPMI culture medium, schizonts were diluted with uninfected erythrocytes for a parasitemia of 3–5% and further supplemented with 2 μm Cpd2 to allow schizont maturation up to the point of egress (22). After 1–4 h at standard culture conditions, cultures were washed at least three times in excess RPMI culture medium to remove Cpd2 and added to 1 volume of Enh1 in RPMI or RPMI only (no drug) for a sample volume of 100 μl at 1% hematocrit. We similarly prepared samples of parasites supplemented with heparin (100 units/ml), a specific inhibitor of erythrocyte invasion (46), to assess the background signal for ring stage parasitemia (see below). We fixed samples (4% paraformaldehyde and 0.0075% glutaraldehyde in PBS (47)) at the outset of the experiment (untreated only) and after 1–2 h of incubation in standard culture conditions (all samples). After extensive washing in PBS and staining with SYBR Green I (1:1000 dilution in PBS; Invitrogen), schizont and ring stage parasitemia were measured by flow cytometry in the FITC channel as described previously (48, 49).

##### T. gondii Invasion Assays

Invasion assays were performed as described previously (20). Briefly, freshly lysed tachyzoites were preincubated for 10 min in varying concentrations of Enh1, keeping the total concentration of DMSO (vehicle) constant in all samples. HFF monolayers, seeded 48 h earlier, were infected at a multiplicity of infection of ∼10, and invasion was allowed to proceed for 10 min at 37 °C. Following fixation, intracellular parasites were enumerated by immunofluorescence and normalized to the number of host cells in a given field.

## Results

### 

#### 

##### Zaprinast Increases Cytosolic Ca^2+^ in a PKG-dependent Manner

**FIGURE 1.**
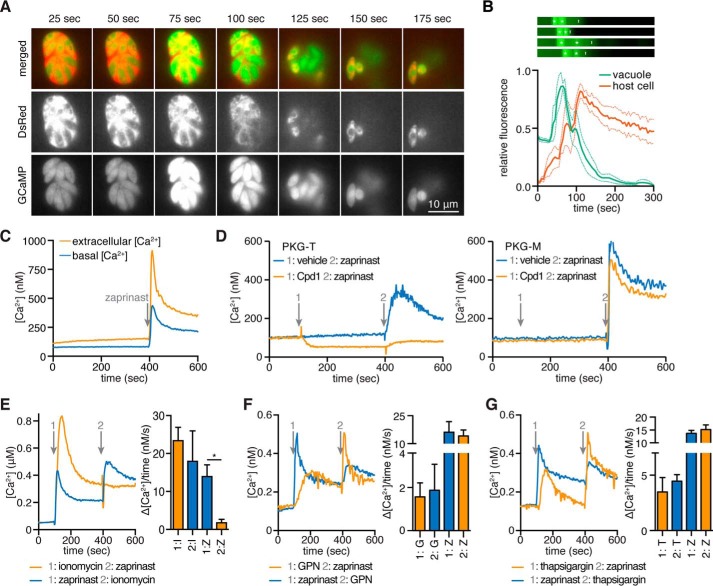
**Zaprinast raises cytosolic Ca^2+^ through the activation of PKG.** *A*, video microscopy of intracellular parasites expressing both GCaMP5 and constitutively secreted DsRed, following the addition of zaprinast at 0 s. *B*, GCaMP5 fluorescence in the region of the parasitophorous vacuole (*green*) or DsRed fluorescence in adjacent areas of the infected host cell (*red*) following the addition of zaprinast. Results shown are mean ± S.E. for four experiments. Kymographs for GCaMP5 fluorescence in all four experiments are shown to indicate times of peak fluorescence (*white asterisks*) relative to the initiation of egress from the region (*white vertical lines*). *C*, intracellular Ca^2+^ concentrations, monitored over time, for wild-type parasites loaded with Fura-2/AM, suspended in buffer containing extracellular (1.8 mm) or basal (100 nm) free Ca^2+^, and stimulated with 100 μm zaprinast at 400 s. *D*, similar measurements were performed for PKG-M and PKG-T parasites loaded with Fura-2/AM and suspended in basal Ca^2+^. Cpd1 or vehicle was added at 100 s, and zaprinast was added at 400 s, as indicated. *E–G*, intracellular calcium concentrations from parasites loaded with Fura-2/AM and then treated with zaprinast, ionomycin, GPN, or thapsigargin at 100 s (*1*) and 400 s (*2*), as indicated. Traces are representative of three independent experiments. *Bar graphs* report the change in cytosolic calcium over 20 s following the addition of each drug. Results shown are mean ± S.E. (*error bars*). *Z*, zaprinast; *I*, ionomycin; *G*, GPN; *T*, thapsigargin; *, *p* < 0.05; one-tailed *t* test.

Previous work has shown that zaprinast triggers *T. gondii* and *P. falciparum* egress in a PKG-dependent manner (20, 22). Because Ca^2+^ is a necessary second messenger during egress (8), we hypothesized that zaprinast might stimulate egress by increasing cytosolic Ca^2+^ in the parasite. To test this, we generated a strain that expressed the genetically encoded Ca^2+^ indicator GCaMP5 (32) in the wild-type *T. gondii* recombinant human background. These parasites were transfected with a plasmid encoding a constitutively secreted fluorescent fusion protein, p30-DsRed, which accumulates in the parasitophorous vacuole before egress (50). This second sensor enabled us to visualize permeabilization of the parasitophorous vacuole, which has been demonstrated to occur via the Ca^2+^-regulated secretion of a perforin-like protein (50). Human fibroblasts infected with recombinant human GCaMP5 were treated with zaprinast and monitored by live video microscopy. Zaprinast elicited a rapid increase in fluorescence compared with the vehicle alone, which was followed by a second, less intense peak of fluorescence (Fig. 1, *A* and *B*). This led to permeabilization of the vacuole membrane, as indicated by diffusion of DsRed, and subsequent egress of the parasites from the host cell (supplemental Video S1).

To determine whether the zaprinast-induced increase in Ca^2+^ represented release of intracellular stores or Ca^2+^ entry, we loaded wild-type parasites with the ratiometric Ca^2+^ indicator Fura-2/AM and stimulated them with zaprinast in buffers containing either a basal Ca^2+^ concentration (100 nm free Ca^2+^) or one resembling the extracellular environment (1.8 mm). We found that cytosolic Ca^2+^ increased in response to zaprinast under both conditions (Fig. 1*C*), although the response was magnified in the presence of extracellular Ca^2+^. This indicates that zaprinast-mediated Ca^2+^ mobilization occurs through release of intracellular stores, which may enhance Ca^2+^ entry, as has been reported for other agonists capable of releasing intracellular stores (9).

Compound 1 (Cpd1), a specific inhibitor of apicomplexan PKG (25), blocks zaprinast-induced egress (20). To determine whether zaprinast's effect on parasite Ca^2+^ depends on PKG, we measured the response to zaprinast after Cpd1 treatment. Sensitivity of apicomplexan PKG to Cpd1 relies on the identity of a residue at the base of the ATP-binding pocket known as the gatekeeper (25). We therefore used strains engineered to express PKG with either the wild-type threonine or a bulky methionine gatekeeper residue that renders PKG refractory to Cpd1 inhibition (31). These strains were loaded with Fura-2/AM, treated with Cpd1 or vehicle, and then stimulated with zaprinast. Parasites treated with vehicle responded to the addition of zaprinast with a sharp Ca^2+^ spike. The addition of Cpd1 did not change the response of the insensitive PKG-M strain. However, the Ca^2+^ spike was severely diminished by Cpd1 in the sensitive PKG-T strain, indicating the need for PKG in this process (Fig. 1*D*). We also observed a slight decrease in the cytosolic Ca^2+^ level of the PKG-T strain after Cpd1 treatment, perhaps indicating that PKG regulates the basal Ca^2+^ level in addition to enhancing it before egress.

##### Zaprinast Mobilizes a Neutral, SERCA-independent Ca^2+^ Store

To further characterize the source of zaprinast-mobilized Ca^2+^, we assessed the involvement of various Ca^2+^ storage organelles. We loaded parasites with Fura-2/AM and suspended them in a buffer containing basal Ca^2+^ and then added the Ca^2+^ ionophore ionomycin. Binding of ionomycin to Ca^2+^ is pH-dependent and falls to negligible levels below pH 7 (51). Ionomycin has therefore been used to specifically mobilize neutral Ca^2+^ stores in both mammalian cells (52) and *T. gondii* (17). We observed a peak in cytosolic Ca^2+^ levels following ionomycin treatment, indicating that Ca^2+^ had been mobilized from neutral stores. Subsequent treatment with zaprinast did not produce an additional Ca^2+^ peak, indicating that the zaprinast-mobilized store had already been depleted by ionomycin. In contrast, treating parasites with zaprinast followed by ionomycin produced a Ca^2+^ spike in response to each compound. These results were corroborated by analyzing the rate of Ca^2+^ release during the 20 s that followed the addition of each compound (Fig. 1*E*). Taken together, these results indicate that the zaprinast-mobilized store comprises a subset of the ionomycin-mobilized stores and as such is predicted to be neutral.

To further rule out acidic Ca^2+^ stores, we treated parasites with GPN before or after the addition of zaprinast. GPN is specifically hydrolyzed in lysosomal compartments, leading to their leakage, as has been shown for the plantlike vacuole in *T. gondii* (18). As predicted by mobilization with ionomycin, the zaprinast-mobilized store was independent from this acidic compartment (Fig. 1*F*).

The ER is the major neutral Ca^2+^ store in many organisms (53). Thapsigargin is an inhibitor of the Ca^2+^ reuptake pump SERCA, which partially localizes to the ER in extracellular *T. gondii* tachyzoites (54). We treated parasites with thapsigargin before or after the addition of zaprinast and found that zaprinast mobilized Ca^2+^ with the same efficiency regardless of the treatment order. The independence of the zaprinast- and thapsigargin-mobilized stores was evident in the rate of Ca^2+^ release following each treatment (Fig. 1*F*). These results indicate either that zaprinast mobilizes a neutral store that is separate from the ER or that the ER is segmented into SERCA-dependent and SERCA-independent Ca^2+^ stores, as has been hypothesized (4).

##### Identifying Small Molecules That Modulate Parasite Ca^2+^

**FIGURE 2.**
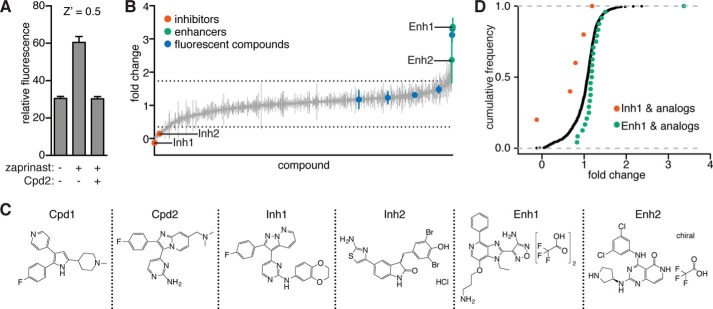
**Compound screen identifies modulators of zaprinast-induced Ca^2+^ signaling.** *A*, GCaMP5-expressing parasites pretreated with Cpd2 or a vehicle control were stimulated with zaprinast or a vehicle control, and fluorescence was measured to determine a *Z*′ score for a zaprinast-based screen for Ca^2+^ modulators. *B*, GCaMP5-expressing parasites were pretreated with 823 compounds from the PKIS libraries. Fluorescence was measured after zaprinast stimulation. Results are -fold change from zaprinast alone after background subtraction. Compounds that fluoresced independently are indicated (*blue*) along with selected enhancers (*Enh*; *green*) and inhibitors (*Inh*; *red*). The mean ± S.D. (*error bars*) of two experiments is shown, and *dashed lines* indicate two S.D. values above and below the mean of all compounds. *C*, structures of Enh1, Enh2, Inh1, and Inh2 as well as the known PKG inhibitors: Cpd1 and Cpd2. *D*, cumulative frequencies of screen values for all compounds (*black*), Inh1 and its analogs (*red*), or Enh1 and its analogs (*green*).

Despite its central importance during infection, few compounds have been demonstrated to specifically interfere with the Ca^2+^ signaling pathways of apicomplexan parasites. Compounds that modulate Ca^2+^ signaling can have clinical value as well as being useful in research. We predicted that the zaprinast response measured in GCaMP5-expressing parasites could form the basis for a phenotypic screen. Such a screen would benefit from the known importance of Ca^2+^ signaling in parasite biology along with the reported success rate of cell-based phenotypic assays. We designed a screen wherein extracellular GCaMP5-expressing parasites were pretreated with compounds for 10 min before stimulation with zaprinast, and parasite fluorescence was measured 4 min later. We expected that compounds that interfered with the zaprinast response would reduce fluorescence. We calculated a *Z*′-factor for this screen to determine its dynamic range and suitability to high throughput screening. To do so, we compared zaprinast-treated parasites to untreated parasites or those pretreated with the PKG inhibitor Compound 2 (Cpd2) (55). These scenarios yielded *Z*′-factors of 0.58 and 0.55, respectively, well above the 0.5 *Z*′-factor considered acceptable for high throughput screening (56). This phenotypic screen is therefore suitable for analyzing large libraries of compounds to identify compounds interfering with parasite Ca^2+^ signaling (Fig. 2*A*).

We obtained the compound libraries published kinase inhibitor sets PKIS1 (57) and PKIS2 (unpublished), from GlaxoSmithKline and applied our phenotypic screen to the 823 ATP mimetics represented in these collections (Fig. 2*B* and supplemental Table S1). The compounds were screened at a single 10 μm dose with two biological replicates. We identified 37 putative inhibitors and 14 putative enhancers, defined as compounds that resulted in fluorescence readings that were more than two S.D. values below or above the mean of all compounds, respectively. Compounds that interfered with accurate measurement of GCaMP5 fluorescence, including two putative enhancers, were identified and excluded from subsequent experiments.

We wondered whether we could identify classes of molecules that inhibit or enhance Ca^2+^ mobilization. To this end, we examined analogs of the most potent enhancer and inhibitor, which we here refer to as Enh1 (PKIS: GSK260205A) and Inh1 (PKIS: GW827099X) (Fig. 2*C*). Compounds with the same core structure as Enh1 generally produced a -fold change in fluorescence of >1 in our screen, indicating that parasite fluorescence was greater in the presence of such compounds than with zaprinast alone (Fig. 2*D* and supplemental Fig. S1). A Kolmogorov-Smirnov test comparing the values obtained from all compounds with those obtained from the Enh1 analogs revealed a significant difference (*p* = 0.0042) (Fig. 2*D*). The combination of a phenyl group at the R1 position and a 3-aminopropyloxy group at the R3 position, as found in Enh1, appeared to produce a particularly effective enhancer; compounds having either group without the other produced only a mild effect. A similar analysis of Inh1 analogs revealed that these compounds tended to inhibit zaprinast-induced Ca^2+^ mobilization (Fig. 2*D* and supplemental Fig. S2). The fluorophenyl group in the R1 position of Inh1 may be responsible for this compound's strong inhibitory effect (as discussed below). This 4-fluorophenyl is also found in Cpd1 and Cpd2. No other R1 side group produced a similarly strong response, although an analogue containing a trifluoromethyl group at the R1 position elicited the second largest response in the group. In summary, although Enh1 and Inh1 are unique within the set of compounds tested for their abilities to augment and repress cytosolic Ca^2+^, their core structures appear to be generally well suited to these purposes. Indeed, further exploration of these scaffolds could lead to the identification of improved modulators of parasite Ca^2+^ signaling.

##### Two Inhibitors Target PKG Using Distinct Chemical Scaffolds

**FIGURE 3.**
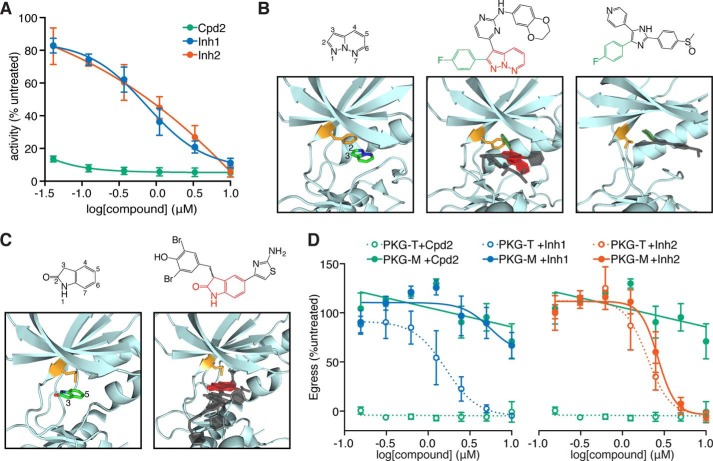
**PKG inhibitors display distinct modes of inhibition.** *A*, activity of recombinant PKG in the presence of increasing concentrations of Inh1, Inh2, or the PKG inhibitor Cpd2. *B*, binding of PP derivatives like Inh1 to protein kinases orients the C2 position toward the gatekeeper residue (*orange*). *Left*, the PP scaffold's orientation for a disubstituted PP in complex with CDK2. *Middle*, the structure of Inh1 is *highlighted* to indicate the PP scaffold (*red*) and the C2 fluorophenyl group (*green*). Two other similar compounds from other kinase structures are *superimposed* over the CDK2 structure in complex with its inhibitor, all showing a similar positioning of the PP scaffold. *Right*, human p38 MAPK with a trisubstituted monocyclic heterocycle oriented similarly as the PP scaffold and extending a fluorophenyl group in the direction of the gatekeeper. *C*, oxindole derivatives bind to protein kinases in a manner that orients their C2 and C5 positions away from the gatekeeper. *Left*, oxindole scaffold of a derivative in complex with NEK2. *Right*, the oxindole scaffold of Inh2. Two other similar compounds from other kinase structures have been *superimposed* on the structure of NEK2 with its inhibitor. *D*, zaprinast-induced egress of parasites carrying Cpd2-sensitive (PKG-T) or resistant (PKG-M) alleles of PKG, following pretreatment with Inh1, Inh2, or Cpd2. Results shown are mean ± S.E. (*error bars*) for *n* = 3 independent experiments.

Because known PKG inhibitors could suppress zaprinast-induced Ca^2+^ release (Fig. 1*D*), we hypothesized that the newly identified compounds could act in a similar manner. We tested the effects of Inh1 and Inh2 (compound GW407323A from the screen) on the *in vitro* activity of recombinant *T. gondii* PKG. Both compounds inhibited PKG activity with IC_50_ values of 580 nm for Inh1 and 670 nm for Inh2 (Fig. 3*A*), suggesting that PKG inhibition is a plausible explanation for their activity. Furthermore, neither inhibitor showed appreciable activity against human PKG or related AGC kinases (57), demonstrating selectivity for the parasite enzyme.

The two inhibitors are derivatives of distinct bicyclic heterocyclic scaffolds with proven activity against protein kinases. Inh1 is a PP with substitutions in the C2 and C3 positions (Fig. 3*B*). Several available kinase structures display a similar orientation of the PP scaffold in the ATP-binding pocket, with the C3 position engaging the hinge region and the C2 position oriented toward the gatekeeper residue. The structures of two such inhibitors complexed with human CDK2 demonstrate the proximity of the C2 position to the bulky phenylalanine-gatekeeper residue (Fig. 3*B*, *middle*). Substitutions at this position decreased the activity of these inhibitors presumably due to steric clash with the gatekeeper (36). The 4-fluorophenyl group in the C2 position of Inh1 is therefore expected to restrict binding of the inhibitor to kinases with relatively small gatekeepers, like the threonine found in *T. gondii* PKG. This type of binding is evident in the structure of p38 MAPK with a ligand similar to Inh1 (SB203580, a monocyclic heterocycle rather than a bicyclic one) that extends the 4-fluorophenyl functional group into the hydrophobic pocket created by a threonine gatekeeper (37) (Fig. 3*B*, *right*). Furthermore, the related human kinase ERK2, normally resistant to SB203580, can be rendered sensitive to this inhibitor by mutation of its gatekeeper glutamine to either an alanine or a threonine (58). This strongly suggests that a small gatekeeper is required to accommodate heterocycles with substitutions similar to those found on Inh1.

Inh2 is an oxindole derivative, with substitutions at the C3 and C5 positions (Fig. 3*C*). Disubstituted and trisubstituted oxindoles are well studied protein kinase inhibitors. Several structures are available of kinases in complex with such inhibitors. In every instance, the oxindole is positioned such that the 2-oxygen and the nitrogen of the bicyclic scaffold engage the hinge residues (Fig. 3*C*, *left*). This configuration leaves the C3 and C5 positions pointing away from the binding pocket. Although the C6 substituent is inward pointing, it does not approach the direction of the gatekeeper. The binding configuration of oxindole derivatives is highly consistent across several different kinase structures, suggesting that Inh2 (a 3,5-disubstituted oxindole) is likely to be gatekeeper-independent.

To determine whether these inhibitors could interfere with other PKG-dependent processes, we next assessed their ability to block zaprinast-induced egress. We directly tested the structural predictions regarding the effect of the gatekeeper on Inh1, but not Inh2, by performing these experiments with the PKG-M and PKG-T strains. We measured zaprinast-induced egress by lactate dehydrogenase release from host cells infected with either strain (Fig. 3*D*). As expected, PKG-T parasites did not egress after Cpd2 treatment, whereas the refractory PKG-M strain egressed similarly to vehicle-treated parasites. Both Inh1 and Inh2 reliably reduced egress relative to vehicle-treated parasites. The similar potencies of these compounds *in vivo* and *in vitro* are consistent with inhibition of PKG being the primary mechanism through which Inh1 and Inh2 block egress (Fig. 3*D*). As predicted from our structural analysis, Inh1 worked in a gatekeeper-dependent manner, reducing egress from cells infected with PKG-T parasites with an IC_50_ of 1.3 μm but leaving PKG-M parasites unaffected. In contrast, Inh2 reduced egress from cells infected with either strain, exhibiting an IC_50_ of 2.5 μm for PKG-T and 3.5 μm for PKG-M. These results were satisfying because we were able to recover inhibitors with distinct scaffolds and modes of inhibiting an enzyme known to be involved in the zaprinast response. Identification of two novel PKG inhibitors through a screen of only 823 compounds demonstrates the power of our approach to identify compounds with biological effects in pathways critical to parasite survival.

##### Two Enhancers Directly Release Intracellular Ca^2+^ Stores

**FIGURE 4.**
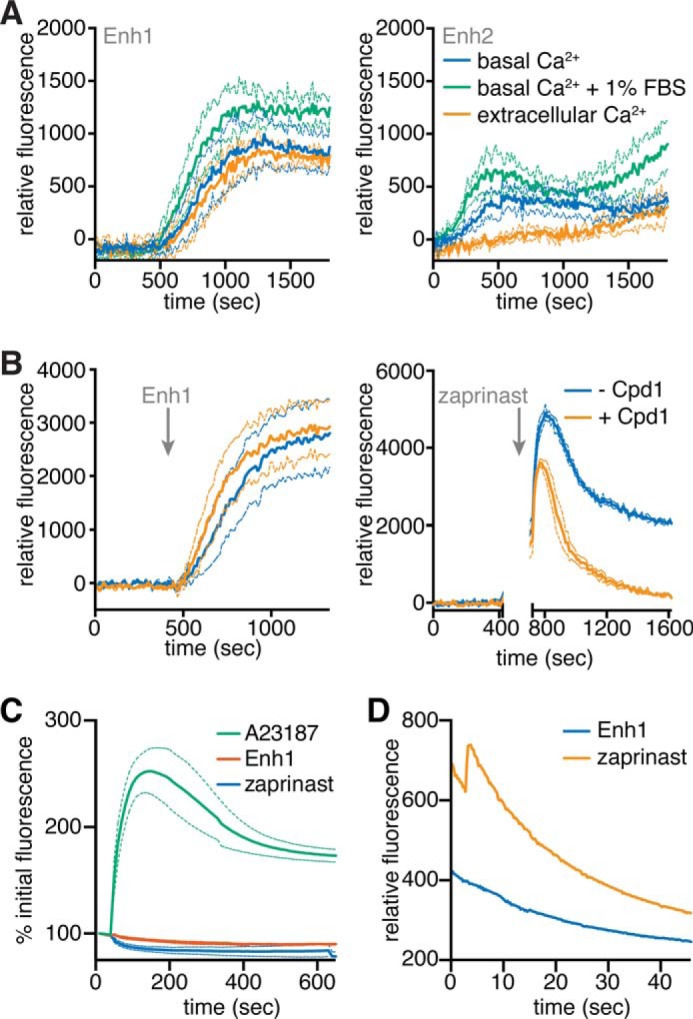
**Enh1 and Enh2 mobilize intracellular Ca^2+^ stores.** *A*, GCaMP6-expressing parasites suspended in buffer supplemented with either extracellular (1 mm) or basal (100 nm) Ca^2+^ concentrations, with or without 1% FBS, and treated with 10 μm Enh1 or Enh2 at time 0. *B*, GCaMP6-expressing parasites treated with 1 μm Cpd1 or vehicle at 100 s and then with 10 μm Enh1 or 100 μm zaprinast at 400 or 750 s, respectively. A *gap* indicates incubation on ice before the addition of zaprinast, in order to capture the peak of the response. Measurements represent fluorescence after the subtraction of background obtained from samples treated with Cpd1 or vehicle, as indicated. Results shown are mean ± S.E. (*error bars*) for *n* = 3 independent experiments. *C* and *D*, intensity of R-GECO-expressing HeLa cells treated with Enh1, zaprinast, or A23187 over 10 min (*C*) or acquired at a faster rate for 1 min (*D*).

Having shown that a screen for modulators of zaprinast-induced Ca^2+^ mobilization reveals novel PKG inhibitors, we turned our attention to Enh1 and another putative enhancer: Enh2, or GSK2188764A (Fig. 2*C*). During the course of this study, the genetically encoded Ca^2+^ indicator GCaMP6f became available. Because GCaMP6f has a broader dynamic range and faster kinetics than GCaMP5 (59), we used GCaMP6f throughout much of this study. We tested whether the enhancers could increase parasite Ca^2+^ independently of zaprinast. Indeed both enhancers could mobilize Ca^2+^ on their own, although they did so with significantly slower kinetics than zaprinast (Fig. 4*A*).

To determine the source of the Ca^2+^ mobilized by the enhancers, we suspended parasites in buffers containing extracellular (1 mm) or basal (100 nm) levels of free Ca^2+^, treated these parasites with 10 μm Enh1 or Enh2, and monitored GCaMP6f fluorescence (Fig. 4*A*). Enh1 increased fluorescence under both conditions, indicating that it mobilizes intracellular stores. Surprisingly, we observed only weak mobilization of Ca^2+^ in response to Enh2 under both conditions. Because the original screen was performed in buffer containing 1% FBS, we tested the effect of this component. The addition of 1% FBS increased Enh2-mediated Ca^2+^ mobilization. We interpreted this to mean that Enh2 requires a cofactor found in FBS. Because the activity of Enh2 is relatively weak and depends on specific conditions, we focused subsequent experiments on Enh1.

We wondered whether, like zaprinast, Enh1 functions through PKG to increase parasite Ca^2+^. Significant overlap in the emission spectra of Enh1 and Fura-2 prevented us from using this ratiometric indicator. However, we were able to use GCaMP6f to make semiquantitative measurements. We recorded fluorescence from GCaMP6f-expressing parasites treated with Cpd1 or vehicle control, followed by stimulation with Enh1 or zaprinast. To record the peak fluorescence induced by zaprinast, we had to incubate parasites on ice before stimulation so as to slow down the response. In contrast to the zaprinast response, Cpd1-treated parasites responded to Enh1 in the same manner as vehicle-treated parasites, indicating that Enh1 does not act through PKG. Although Cpd1 accelerated the return to basal Ca^2+^ levels following zaprinast treatment, it only partially decreased the initial Ca^2+^ spike induced by zaprinast (Fig. 4*B*). This is reminiscent of results obtained from Fura-2-loaded parasites, where the increase in Ca^2+^ following zaprinast could never be fully suppressed by PKG inhibition (Fig. 1*D*).

We wondered whether zaprinast or Enh1 could induce Ca^2+^ fluxes in mammalian cells. We transfected HeLa cells with the plasmid CMV-R-GECO (42), which encodes a variation of the GCaMP Ca^2+^ indicators under the mammalian CMV promoter. We treated these transfected cells with zaprinast, Enh1, or, as a positive control, the Ca^2+^ ionophore A23187. Video microscopy revealed that R-GECO-expressing HeLa cells increased fluorescence in response to A23187 but that neither zaprinast nor Enh1 produced a significant change (Fig. 4*C*). Given that zaprinast is known to inhibit phosphodiesterases in mammalian cells (60), we considered that these drugs could be having a subtle effect that was below our limit of detection. We therefore repeated the assay with greater spatial and temporal resolution. We observed a slight increase in R-GECO fluorescence in response to zaprinast during the first 10 s of treatment. However, Enh1 had no effect on host cell Ca^2+^ (Fig. 4*D*), indicating that the effects of Enh1 are specific to Ca^2+^ signaling in the parasite.

##### Enh1 Inhibits T. gondii Survival

**FIGURE 5.**
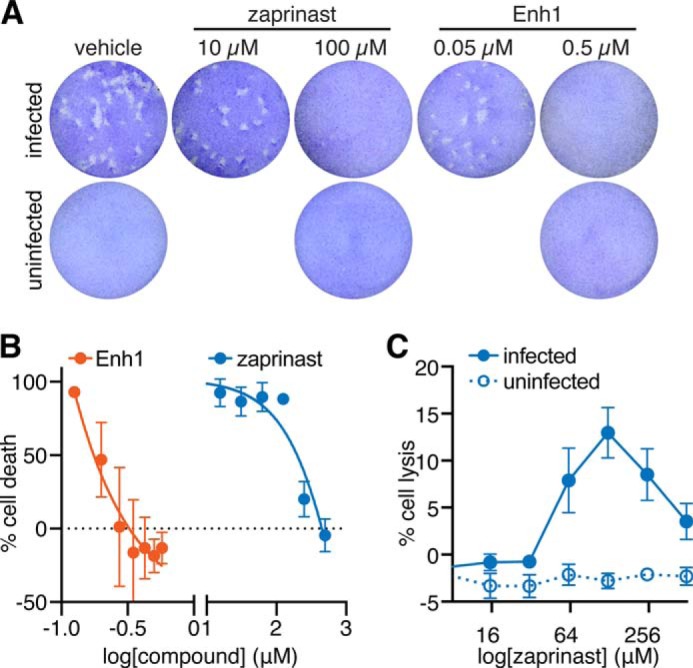
**Enhancers of Ca^2+^ mobilization show antiparasitic activity.** *A*, plaque formation in the presence of the indicated concentrations of Enh1 or zaprinast. The drug concentrations indicated did not affect host cell survival. *B*, dose-dependent effect of Enh1 and zaprinast on parasite viability, assayed by monolayer disruption, 3 days postinfection, at the indicated drug concentrations. Results shown are mean ± S.E. (*error bars*) for *n* = 3 independent experiments. *C*, host cell lysis following 1 h of infection in the presence of varying zaprinast concentrations.

Zaprinast and related phosphodiesterase inhibitors were recently shown to block *T. gondii* proliferation in human fibroblasts (61). Altering Ca^2+^ levels using ionophores has also been shown to affect parasite viability (62). We therefore hypothesized that Enh1 would similarly inhibit survival of *T. gondii*. We tested the effect of these compounds using plaque assays as an indication of parasite survival following treatment with Enh1 or zaprinast. Plaque formation was inhibited by zaprinast and Enh1 at 100 and 0.5 μm, respectively, whereas 10-fold lower concentrations allowed normal plaque formation similar to vehicle alone (Fig. 5*A*). These results for zaprinast agree with previously published work (61). In order to determine the inhibitory concentrations of these compounds more precisely, we performed lytic assays in which host cells were exposed to parasites pretreated with varying concentrations of zaprinast or Enh1, and the degree of host cell lysis was assessed by crystal violet staining of the monolayer, 3 days later. Enh1 showed a steep dose response, with 350 nm completely inhibiting parasite-induced cell death, and an EC_50_ of 180 nm. In contrast, 500 μm zaprinast was required to cause complete inhibition of parasite-induced cell death, and the IC_50_ of zaprinast was 200 μm (Fig. 5*B*).

We noticed the discrepancy between the amount of zaprinast required to kill *T. gondii* in plaque assays (100 μm) and the amount needed in lytic assays (500 μm). Because a rise in cytosolic Ca^2+^ stimulates parasite motility (2), we wondered if the movement of *T. gondii* in response to concentrations between 100 and 500 μm might mechanically wound and kill host cells, which would be indistinguishable from parasite survival in the lytic assay. We tested this hypothesis by incubating host cell monolayers with parasites pretreated with various concentrations of zaprinast and then measuring lactate dehydrogenase release from host cells. We found that treatment with 64–510 μm zaprinast resulted in host cell lysis, with a maximal effect at 130 μm. Host cell lysis was not observed with zaprinast in the absence of *T. gondii* (Fig. 5*C*). Due to the lower multiplicity of infection used in plaque assays compared with lytic assays, host cells in plaque assays would be unlikely to experience observable cell wounding. We therefore expect the plaque assays to provide a more accurate measure of zaprinast's antiparasitic activity.

##### Enh1 Induces Asynchronous Ca^2+^ Fluxes

**FIGURE 6.**
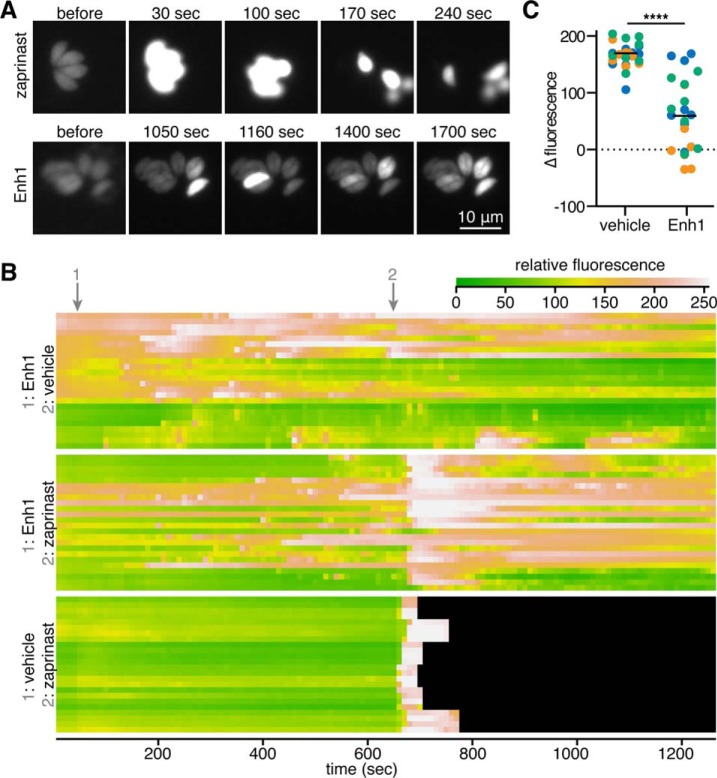
**Enh1 elicits asynchronous cytosolic Ca^2+^ fluxes and blocks zaprinast-induced egress.** *A*, video microscopy of GCaMP6f-expressing parasites treated with Enh1 or zaprinast. Time after the addition of the compound is indicated. Different times were used to capture the fast and slow responses of zaprinast and Enh1, respectively. *B*, kymographs illustrate average fluorescence intensities of individual parasites, per *row*, during the course of the treatment indicated. *Black* indicates that parasites egressed from vacuoles. *C*, change in fluorescence of the parasites illustrated in *B* over the 40 s following the addition of zaprinast. Measurements from each biological replicate are *colored* separately. Mean change for each group is indicated with a *horizontal line*. ****, *p* < 0.0001, two-tailed *t* test.

We thought it likely that the plaquing defect caused by Enh1 was related to the Ca^2+^ disregulation induced by this compound. We therefore characterized the Ca^2+^ response to Enh1 in more detail. We recorded videos of intracellular GCaMP6f-expressing parasites treated with Enh1 and compared the result with zaprinast treatment, which we have shown induces egress. We observed GCaMP6f activity in both cases, indicating that Enh1 can act on intracellular parasites. However, the profiles of zaprinast and Enh1-mediated Ca^2+^ mobilization were remarkably different. Whereas zaprinast induced a fast, strong Ca^2+^ peak (Fig. 6*A* and supplemental Video S2), Enh1 elicited a slow, asynchronous effect in which some parasites appeared to experience multiple Ca^2+^ fluxes of similar magnitudes over the course of several minutes (Fig. 6*A* and supplemental Video S3). The response to these compounds was similar in extracellular parasites (data not shown).

We generated kymographs illustrating the responses of individual GCaMP6f parasites to Enh1. Wondering whether pretreatment with Enh1 would affect the ability of parasites to respond to other Ca^2+^ agonists, we treated some of these parasites with zaprinast as well (Fig. 6*B*). The sporadic flashes of Ca^2+^ triggered by Enh1 were recapitulated, and all vehicle-treated parasites experienced an increase in Ca^2+^ immediately after the addition of zaprinast. However, Enh1-treated parasites varied in their responses to zaprinast, exhibiting asynchronous Ca^2+^ fluxes and inconsistent magnitudes in their Ca^2+^ increases. In rare cases, Ca^2+^ concentrations in Enh1-treated parasites even dropped upon zaprinast treatment. We quantified the changes in fluorescence of individual parasites over the 40 s following zaprinast addition and observed a more varied and overall diminished response to zaprinast in Enh1-treated parasites (Fig. 6*C*). Taken together, these results suggest that Enh1 may partially deplete the Ca^2+^ stores mobilized by zaprinast.

##### Enh1 Blocks Ca^2+^-related Phenotypes in T. gondii and P. falciparum

**FIGURE 7.**
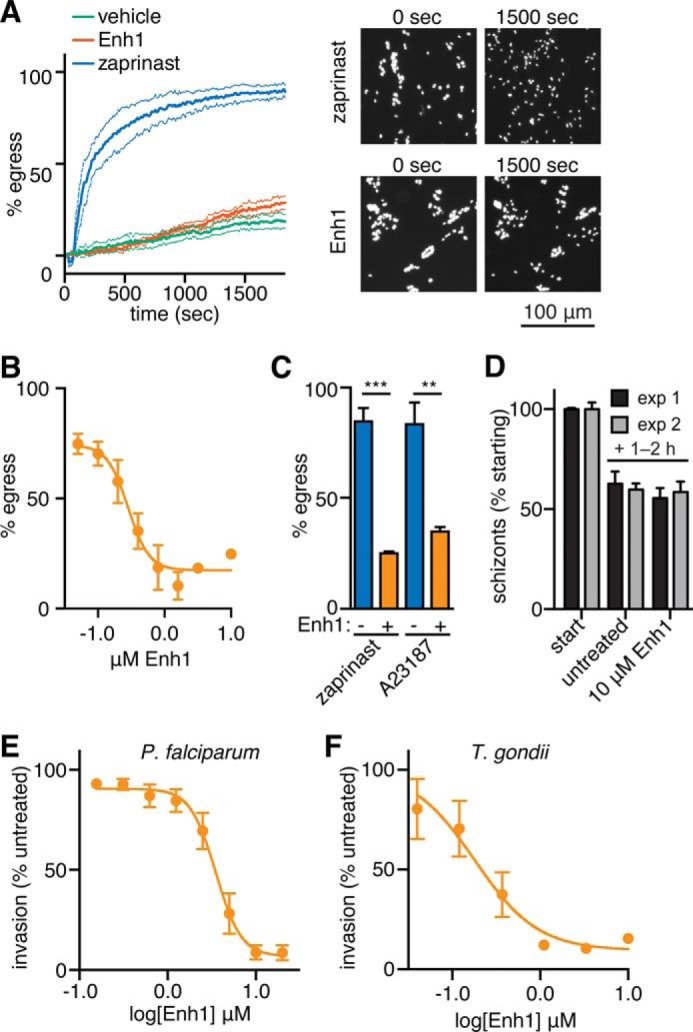
**Enh1 blocks egress Ca^2+^-related phenotypes in *T. gondii* and *P. falciparum*.** *A*, egress of intracellular parasites treated with zaprinast, Enh1, or a vehicle control. The number of intact vacuoles was monitored by live microscopy over 30 min. Representative images before and after treatment are shown. *B*, dose-dependent inhibition of zaprinast-induced egress following pretreatment with Enh1 or vehicle. *C*, Enh1 inhibition of egress induced by either zaprinast or A23187. Results shown are mean ± S.E. (*error bars*) for *n* = 3 independent experiments. ***, *p* < 0.001; **, *p* < 0.01. *D–E*, schizonts were released from Cpd2 arrest immediately preceding the addition of Enh1. *D*, after 1–2 h, the remaining schizonts (mean ± S.D. for three technical replicates) were counted and normalized to their initial abundance (3.6 and 5.4% in each experiment, respectively). *E*, Enh1 blocks invasion of erythrocytes by *P. falciparum*, measured 1–2 h following release from Cpd2, as assessed from the ring stage parasitemia. Mean invasion ± S.E. is expressed as a percentage of invasion without drug (9.5 and 8.1% in each experiment, respectively). Background was assessed using heparin as a specific blocker of invasion and was comparable with the signal observed with saturating concentrations of Enh1. *F*, dose-dependent inhibition of *T. gondii* invasion following 10-min pretreatment of parasites before invasion. Results shown are mean ± S.E. for *n* = 3 independent experiments.

While examining Enh1-treated parasites, we noticed that this compound did not induce egress and in fact suppressed egress in response to zaprinast (Fig. 6*C*). To confirm these effects, we treated intracellular GFP-expressing parasites with Enh1 and quantified the number of intact vacuoles at various times after treatment, using fluorescence to monitor the infection using automated image analysis. This assay is capable of directly measuring egress for hundreds of vacuoles per sample. Surprisingly, despite its effects on extracellular parasites, Enh1 failed to stimulate egress beyond the spontaneous egress observed in the vehicle control. In contrast, zaprinast treatment resulted in ∼90% of vacuoles egressing within 30 min (Fig. 7*A*), as described previously (20). We then examined the effect of a 10-min Enh1 pretreatment and found that it robustly blocked zaprinast-induced egress. Analysis of the dose-dependent inhibition of egress by Enh1 revealed an EC_50_ of 290 nm, within the range of concentrations that inhibit plaque formation (Fig. 7*B*). These results suggest that the antiparasitic activity of Enh1 is mediated by its inhibition of parasite egress.

We wondered whether we could generalize the Enh1-associated egress defect beyond zaprinast-induced egress. We therefore tested the ability of Enh1 to block egress induced by the Ca^2+^ ionophore A23187. Enh1 also produced a significant block on A23187-induced egress (Fig. 7*C*), demonstrating that its effects on parasite Ca^2+^ signaling extend to a variety of agonists.

We tested whether Enh1 also affects other apicomplexan parasites that use Ca^2+^-based signal transduction. Asexual, blood stage *Plasmodium* parasites undergo Ca^2+^-dependent egress from infected erythrocytes, followed by Ca^2+^-dependent invasion into new erythrocytes (6, 63). To assess the effect of Enh1 on these processes at the relevant developmental stage, we allowed purified schizonts to complete their intracellular maturation while blocked with Cpd2 from rupturing and egressing (22). We administered Enh1 to parasites immediately following washout of Cpd2. After allowing 1–2 h of incubation with the compound, we measured egress and reinvasion using a flow cytometry-based assay that distinguishes schizonts from recently invaded ring stage parasites (49). Whereas Enh1 does not reduce parasite egress at concentrations up to at least 10 μm (Fig. 7*D*), the compound completely blocks invasion within the tested range (IC_50_ = 3.2 μm) (Fig. 7*E*). Wondering whether Enh1 could also inhibit invasion in *T. gondii*, we incubated tachyzoites with varying doses of Enh1 for 20 min and tested their ability to infect host cells. As in *P. falciparum*, Enh1 strongly inhibited *T. gondii* invasion (Fig. 7*F*). Furthermore, the IC_50_ of Enh1 in the invasion assay was 180 μm, similar to that in the egress assay, arguing for a common mechanism for the effect of Enh1 on both Ca^2+^-related phenotypes. In summary, Enh1 modulates Ca^2+^-dependent processes in diverse apicomplexan parasites.

## Discussion

Ca^2+^ signaling plays a central role in apicomplexan biology, yet few of the components that regulate Ca^2+^ uptake and release have been identified. In this study, we extend our understanding of these processes by demonstrating that PKG activity is needed for the robust Ca^2+^ response elicited by phosphodiesterase inhibitors. Furthermore, we determine the source of Ca^2+^ to be distinct from other previously described stores. We use this phenomenon as the basis of a phenotypic screen that allowed us to identify several novel inhibitors and enhancers of Ca^2+^ signaling. Two of the inhibitors could be shown to interfere with Ca^2+^ signaling by specifically targeting parasite PKG. In contrast, the enhancers could be shown to increase Ca^2+^ independently from zaprinast and in fact prevented parasite egress by apparently depleting intracellular Ca^2+^ stores. This compound displayed antiparasitic properties against both *T. gondii* and *P. falciparum*, establishing a new mechanism for interfering with apicomplexan parasitism.

The signaling events that trigger parasite egress remain poorly defined. Ca^2+^ ionophores have long been known to stimulate egress in *T. gondii* (64). However, more recently, phosphodiesterase inhibitors were shown to have similar effects (20, 61). Our experiments revealed a strong and rapid release of Ca^2+^ in response to zaprinast treatment through the use of both the established Ca^2+^ indicator Fura-2 and the newly adapted genetically encoded sensors GCaMP5 and GCaMP6f (29). We provide conclusive evidence linking PKG to the zaprinast-induced increase in cytosolic Ca^2+^, using a chemical-genetic approach to demonstrate specific inhibition of PKG by Cpd1 and Cpd2. Sensitivity to both inhibitors depends on the relatively small gatekeeper of apicomplexan PKGs, which we mutated to a methionine that preserves kinase activity but renders PKG refractory to inhibition (31). The changes in the zaprinast response caused by Cpd1 can therefore be fully attributed to PKG because no such changes were observed upon treatment of the resistant strain (PKG-M). However, comparing the response to zaprinast in parasites pretreated with Cpd1 with those pretreated with vehicle shows that the initial sharp Ca^2+^ peak induced by zaprinast is incompletely suppressed by Cpd1, in contrast to its complete inhibition of zaprinast-induced egress (20). Suppression of this peak appeared greater when assayed using Fura-2 (Fig. 1*D*) than when using GCaMP6 (Fig. 4*B*). This may result from the semiquantitative nature of GCaMP6 and the potentially non-linear relationship between fluorescence and Ca^2+^ concentration. Additionally, differences in the subcellular distribution of the two indicators may influence their responses to different sources of Ca^2+^. Ratiometric measurements with Fura-2 also revealed that inhibition of PKG by Cpd1 decreased basal Ca^2+^ concentrations in extracellular parasites, suggesting that PKG might also be necessary to maintain resting levels of Ca^2+^ in extracellular parasites. These changes in basal Ca^2+^ concentrations might not be evident with GCaMP6f due to its higher *K_d_* (375 nm) (59) compared with that of Fura-2 (135 nm) (43).

We characterized the zaprinast-mobilized store as neutral because it can be depleted by ionomycin. However, we found that this store is independent of the thapsigargin-mobilizable store. This is surprising, given that the ER is the only neutral Ca^2+^ store that has been characterized in *T. gondii*. SERCA, the target of thapsigargin, localizes to the ER in intracellular parasites but redistributes so as to only partially colocalize with the ER in extracellular parasites (54). Because our experiments were done in extracellular parasites, it is possible that zaprinast mobilizes Ca^2+^ from a section of the ER lacking SERCA under these conditions, as previously suggested for the ethanol-mobilized Ca^2+^ stores (65). In *P. falciparum*, zaprinast has been shown to work through *P. falciparum* PKG to trigger changes in the levels of various precursors of the second messenger IP_3_. Presumably, this increases IP_3_, which then interacts with the IP_3_ receptor to stimulate release of Ca^2+^ from intracellular stores (19). An IP_3_ receptor has not been identified in apicomplexans, but treating parasites with ethanol raises levels of IP_3_ and stimulates Ca^2+^ release, providing evidence for the presence of such a channel (66). Our results therefore indicate that zaprinast functions, at least in part, through *T. gondii* PKG and probably mobilizes a neutral, SERCA-independent, IP_3_ receptor-gated store.

Genetically encoded calcium indicators provide excellent reproducibility and circumvent many problems associated with loading and compartmentalization of chemical probes. Here we demonstrate that such indicators can be used to identify compounds that alter apicomplexan Ca^2+^ signaling and its dependent processes, such as egress and invasion. The simplicity and robustness of this cell-based phenotypic screen, with a *Z*′-factor >0.5, makes it compatible with high throughput screening efforts. As proof of concept, we screened a library of 823 ATP mimetics from GlaxoSmithKline for compounds that could alter the zaprinast-induced increase in cytosolic Ca^2+^. Identification of two PKG inhibitors, with distinct chemical scaffolds and mechanisms of inhibition, validated our screen and demonstrates the power of the approach. Unexpectedly, our screen also revealed two compounds that, when used in combination with zaprinast, augmented Ca^2+^ levels. One such compound, Enh1, elicited repeated cycles of Ca^2+^ increase and decrease with overall cytosolic Ca^2+^ building at a population level. These repeated cycles are reminiscent of what others have seen when Fluo-4/AM-loaded parasites were treated with thapsigargin (54), perhaps indicating that Enh1 also inhibits Ca^2+^ uptake pathways.

In contrast to zaprinast and Ca^2+^ ionophores, Enh1 failed to stimulate egress despite raising cytosolic Ca^2+^ levels. In fact, Enh1 blocked the ability of these agonists to stimulate parasite egress. Furthermore, treatment with Enh1 blocked tachyzoite invasion and plaque formation at concentrations similar to those required to block egress. These results suggest that Enh1 depletes essential intracellular Ca^2+^ stores, which have been previously suggested to mediate invasion and egress. Consistent with this view, we observed diminished changes in GCaMP fluorescence in response to zaprinast, following Enh1 treatment (Fig. 6*C*). However, without a complete understanding of Enh1 function, we cannot rule out the possibility that inhibition of egress may be independent of its ability to modulate Ca^2+^ because (i) some parasites in which Enh1 did not elicit Ca^2+^ fluctuations still failed to egress in response to zaprinast, and (ii) A23187, which should equilibrate Ca^2+^ across membranes, failed to overcome Enh1 inhibition. Compounds similar to Enh1 have been shown to inhibit mammalian kinases belonging to the AGC family (67). Having already established that *T. gondii* PKG, a member of the AGC kinase family, mediates Ca^2+^ release, it is an intriguing possibility that a related kinase might oppose its activity.

Enh1 robustly inhibited the ability of *P. falciparum* to invade erythrocytes, although it did not affect egress. Despite the many parallels between egress of *P. falciparum* and *T. gondii*, substantial differences have also been uncovered. *Plasmodium* egress is a fast, highly synchronized process, dependent on a cascade of proteolytic activity (68). Genetic evidence supports this distinction with mutants in the calcium-responsive protein DOC2.1 blocking both egress and invasion in *T. gondii* but only invasion in *P. falciparum* (69), mirroring the effects of Enh1. The intracellular Ca^2+^ stores of *Plasmodium* have not been investigated as thoroughly as those of *T. gondii*, but there is some evidence that it is not solely dependent on its intracellular Ca^2+^ stores and in fact utilizes Ca^2+^ found within the parasitophorous vacuole (70). In light of this, the differential responses of *P. falciparum* and *T. gondii* to Enh1 are perhaps not surprising. That Enh1 blocks invasion of *P. falciparum* demonstrates this compound's ability to perturb Ca^2+^-dependent processes across multiple members of the Apicomplexa.

Resistance to front line antimalarials is increasing (71, 72), and new treatment options are needed. Rational design strategies can identify drugs with minimal off-target effects but focus on a limited repertoire of signaling pathways. The *Toxoplasma* Ca^2+^ signaling network shows evidence of conservation within apicomplexans while being dissimilar to human signaling pathways. By screening for modulation of this pathway, we have prioritized compounds that are likely to interfere selectively with infection without focusing on a single parasite protein. This screen can be performed with commonly available equipment and should be easily scalable to larger collections of compounds. Our success in identifying new modulators of Ca^2+^ signaling with effects that extend to multiple apicomplexans highlights the power of such an approach. In particular, Enh1 inhibits parasite viability with an EC_50_ in the nanomolar range and provides a good lead for the development of antiparasitic compounds. Further characterization of these compounds may identify novel components of apicomplexan Ca^2+^ signaling pathways and improve our ability to target these pathways specifically.

## Author Contributions

S. M. S., M. A. H. T., and A. S. P. designed and conducted the experiments and analyzed the data. S. M. S. wrote the majority of the manuscript, with specific sections contributed by M. A. H. T. and A. S. P. C. G. H. constructed the *T. gondii* strains with different PKG alleles. M. E. B., R. H., and W. J. Z. provided key reagents and advice. F. T. and N. J. W. synthesized Cpd2. M. T. D., S. N. J. M., and S. L. supervised the work in their respective laboratories and contributed to the analysis of experiments and writing of the manuscript.

## Supplementary Material

Supplemental Data

## Footnotes

2 The abbreviations used are:

ER: endoplasmic reticulum
GPN: l-phenylalanine-naphthylamide
IP_3_: inositol 1,4,5-triphosphate
PKG: protein kinase G
HFF: human foreskin fibroblast
PP: pyrazolopyridazine
Cpd1 and Cpd2: compound 1 and 2, respectively
Enh1 and Enh2: enhancer 1 and 2, respectively
Inh1 and Inh2: inhibitor 1 and 2, respectively.
