# Supplementary material for: Using a Genetically Encoded Sensor to Identify Inhibitors of Toxoplasma gondii Ca2+ Signaling
Source: J Biol Chem. 2016 Mar 1;291(18):9566–80. doi: 10.1074/jbc.M115.703546 (PMC4850295; doi:10.1074/jbc.M115.703546)
Supplement: Supplemental Data [file supp_291_18_9566__index.html]

Using a Genetically Encoded Sensor to Identify Inhibitors of Toxoplasma gondii Ca2+ Signaling — Identifying Modulators of Apicomplexan Ca2+ Signaling — Supplemental Data 

# Using a Genetically Encoded Sensor to Identify Inhibitors of *Toxoplasma gondii* Ca2+ Signaling

## Supplemental Data

**Files in this Data Supplement:**

- Fig. S1, Fig. S2 - Figure S1. Structures and activities of compounds related to Enh1. Figure S2. Structures and activities of compounds related to Inh1.
- Table S1 - Complete results from the compound screen. GCaMP5-expressing parasites were pre-treated with 823 compounds from the PKIS libraries. Fluorescence was measured after zaprinast stimulation. Fold change from zaprinast alone was calculated after background subtraction. Standard deviation was calculated from the two independent experiments. Relative fluorescence of each compound, measured in the absence of parasites, is listed.
- Video S1 - Video microscopy of intracellular parasites expressing both GCaMP5 and constitutively secreted DsRed, following the addition of zaprinast at 0 sec.
- Video S2 - Video-microscopy of GCaMP6f-expressing parasites treated with 100 ?M zaprinast. Time after the addition of the compound is indicated.
- Video S3 - Video-microscopy of GCaMP6f-expressing parasites treated with 10 ?M Enh1. Time after the addition of the compound is indicated.
