# Supplementary material for: Using a Genetically Encoded Sensor to Identify Inhibitors of Toxoplasma gondii Ca2+ Signaling
Source: J Biol Chem. 2016 Mar 1;291(18):9566–80. doi: 10.1074/jbc.M115.703546 (PMC4850295; doi:10.1074/jbc.M115.703546)
Supplement: Supplemental Data [file supp_M115.703546_suppfigs.pdf]

Figure S1. Structures and activities of compounds related to Enh1.

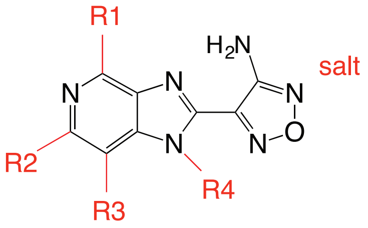

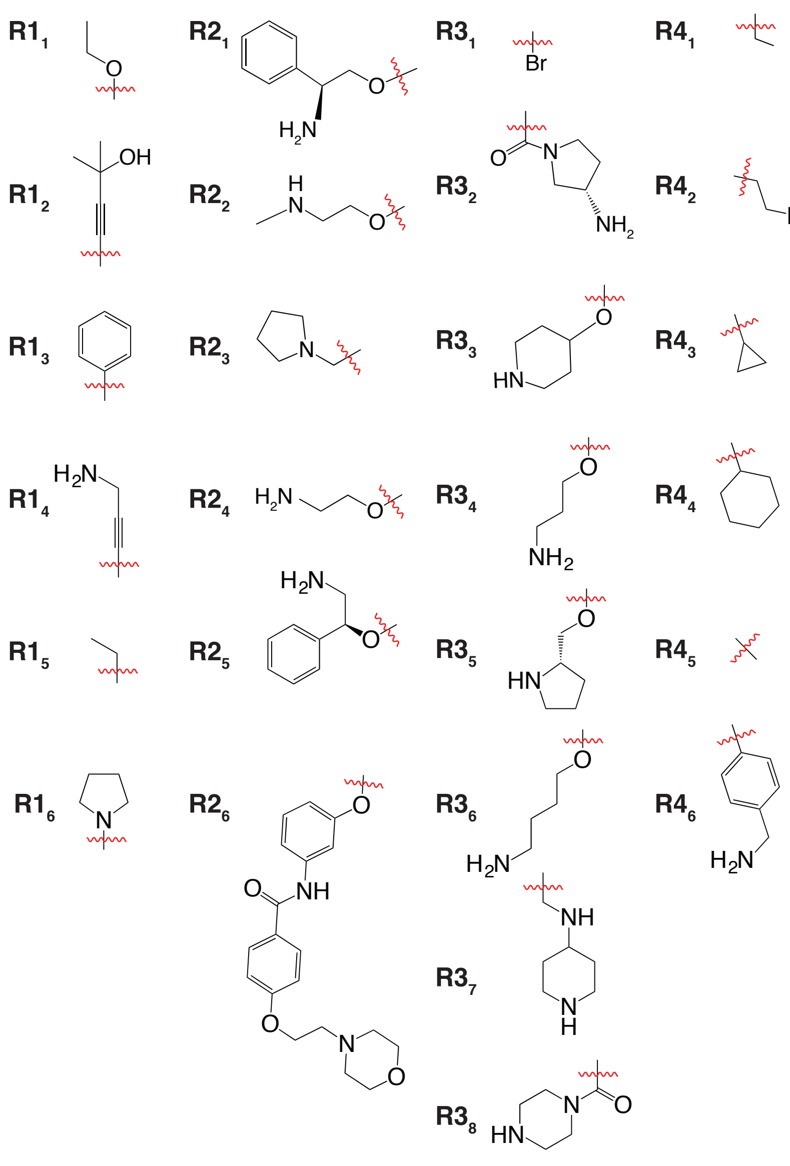

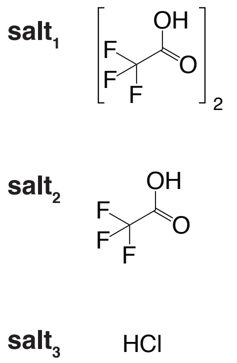

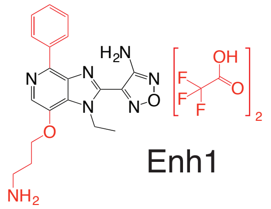

Compound	R1	R2	R3	R4	Salt	Score
SB-738004	-	-	1	1	-	0.830
SB-737198	1	-	-	1	-	0.845
GSK-938890A	2	-	-	1	-	1.022
GSK943949A	2	1	1	1	-	1.114
SB-772077-B	-	-	2	1	3	1.135
GSK949675A	2	2	-	1	3	1.152
GSK1000163A	2	3	-	1	-	1.153
GSK619487A	2	-	3	1	2	1.184
GSK902056	2	4	-	1	-	1.153
SB-751399	3	-	-	2	-	1.355
GSK561866B	4	-	4	1	2	1.218
GSK614526A	2	-	5	1	2	1.198
GSK1007102B	2	5	-	1	2	1.188
SB-736302	-	-	-	3	-	1.283
SB-734117	-	-	-	4	-	1.214
GSK507274A	2	-	6	1	-	1.063
SB-747651-A	-	-	7	1	-	1.501
SB-750140	-	-	-	6	-	1.416
SB-759335-B	-	-	8	1	3	1.333
GSK269962B	-	6	-	1	3	1.303
SB-736290	-	-	-	5	-	1.181
SB-751148	5	-	-	1	-	1.115
SB-744941	6	-	-	1	-	0.961

Figure S2. Structures and activities of compounds related to Inh1.

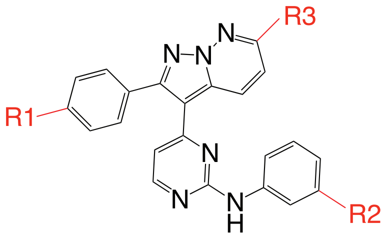

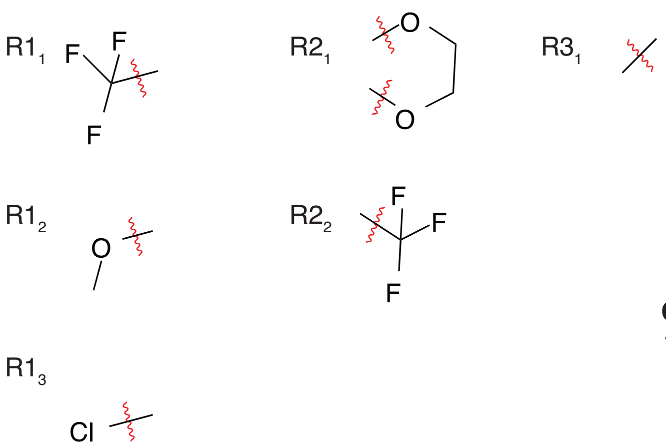

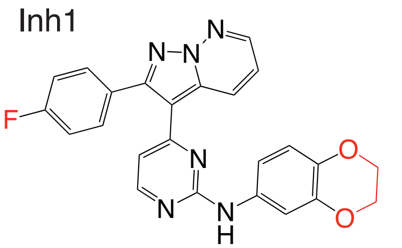

Compound	R1	R2	R3	Score
GW832467X	1	1	1	0.666
GW827105X	2	2	-	0.796
GW827106X	2	1	-	0.989
GW829055X	3	-	-	1.188
